# Renin-Angiotensin Blockade Reduces Readmission for Acute Chest Syndrome in Sickle Cell Disease

**DOI:** 10.7759/cureus.23567

**Published:** 2022-03-28

**Authors:** Nneoma Wamkpah, Anuj Shrestha, Gary Salzman, Stephen Simon, Sahil Suman, Alan Poisner, Agostino Molteni

**Affiliations:** 1 Otolaryngology–Head and Neck Surgery, Washington University School of Medicine, Saint Louis, USA; 2 Hematology and Oncology, University of Missouri–Kansas City, Kansas City, USA; 3 Internal Medicine, University of Missouri–Kansas City, Kansas City, USA; 4 Bioinformatics, University of Missouri–Kansas City, Kansas City, USA; 5 Pharmacology, University of Missouri–Kansas City, Kansas City, USA; 6 Pathology, University of Missouri–Kansas City, Kansas City, USA

**Keywords:** pulmonary fat embolism (pfe), angiotensin ii receptor blockers (arb), angiotensin converting enzyme inhibitors (acei), sick sickle cell disease (scd), acute chest syndrome (acs)

## Abstract

Rationale

Acute chest syndrome (ACS) is a life-threatening complication of sickle cell disease (SCD). Current treatment is supportive-supplemental oxygen, transfusions, and antibiotics. Prevention of ACS may reduce morbidity and mortality in patients with SCD.

Acute chest syndrome appears similar to pulmonary fat embolism (PFE), a complication of severe skeletal trauma or orthopedic procedures from pulmonary micro-vessel blockage by bone marrow fat. Vascular obstruction and bone marrow necrosis occur in PFE and ACS.

Pulmonary fat embolism rat models have shown that angiotensin-converting enzyme inhibitors (ACEI) and angiotensin II receptor blockers (ARB) mitigate damage in PFE. These medications could work similarly in ACS. We hypothesize that time to readmission after one hospitalization for ACS will be reduced in patients taking ACEI or ARB compared to patients who are not.

Methods

This is a retrospective cohort study. Inclusion criteria are adults (18 to 100 years) with sickle cell anaemia (HbSS), hemoglobin SC (HbSC) disease, sickle cell thalassemia (HbSβThal), hospitalized with ACS over 16 years (January 1, 2000, to March 31, 2016); patients who take and don’t take ACEI or ARB. Children (<18 years old), elderly adults (>100 years old), pregnant patients, and patients with sickle cell trait were excluded.

Data was collected from the Health Facts database, which contains de-identified information from the electronic medical records of hospitals in which Cerner© has a data use agreement.

Kaplan-Meier estimates explored a time-to-event model of ACS readmission. Multivariable analysis (age, gender, smoking history) was conducted using Cox proportional hazards regression. Results were reported around a 95% confidence interval.

Results

There were 6972 patients in total. Of which, 9.6% (n = 667) reported taking ACEI or ARB. Results for the covariates were: average age of 38 years old; 63% female (n = 4366/6969); 16% smokers (n = 1132).

Readmission rates were higher for patients not taking ACEI/ARB than those who did: 0.44 (95% CI 0.43, 0.46) versus 0.28 (95% CI 0.24, 0.31) at one year, and 0.56 (95% CI 0.55, 0.58) versus 0.33 (95% CI 0.29, 0.37) at two years. Age had the strongest effect on readmission rates for patients taking ACEI/ARB (adjusted hazards ratio 0.78 [95% CI 0.68, 0.91]).

Conclusion

Patients with SCD who reported taking ACEI or ARB had lower readmission rates for ACS; age was the strongest covariate. Our results may have a significant impact on the prevention of ACS. Prospective studies comparing ACEI or ARB therapy versus placebo are needed to confirm this preventative effect.

## Introduction

Sickle cell disease (SCD) manifests from the inheritance of two copies of a mutant gene. The mutation GAG to GTG, substitutes valine for glutamic acid at position 6 in the beta-globin chain of hemoglobin A, resulting in abnormal hemoglobin called hemoglobin S [1-3]. SCD is one of the most common autosomal recessive disorders in the world. Approximately 8% of African Americans are heterozygous for the disease, meaning they have the sickle cell trait, whereas approximately 1 in 600 is homozygous and have sickle cell disease [4]. Hemoglobin S polymerizes on deoxygenation and the polymers make the erythrocyte rigid, distort its shape, and cause structural damage in the red-cell membrane, all of which alter the rheologic properties of the cell, impair blood flow through the microvasculature and lead to hemolysis and vaso-occlusive episodes [5].

The two most common acute events seen in SCD are vaso-occlusive pain crisis caused by physical and adhesive entrapment of red cells containing hemoglobin S in the microcirculation, and acute chest syndrome (ACS), a lung injury syndrome [6-7]. Pulmonary complications such as ACS and pulmonary hypertension are major causes of morbidity and mortality in SCD [4]. Symptoms are non-specific and include chest pain, tachypnea, fever, cough, and arterial oxygen desaturation. Acute chest syndrome is responsible for 25% of all deaths in SCD [8-9].­­

Three major causes of ACS have been proposed: pulmonary infection, embolization of bone marrow fat, and intravascular pulmonary sequestration of sickled erythrocytes resulting in lung injury and infarction [4]. Pulmonary fat embolism (PFE), the blockage of pulmonary microvasculature by bone marrow fat, causes a more severe and distinct form of ACS via vascular obstruction and bone marrow necrosis [10-12]. The bone marrow undergoes necrosis and its contents including fat, are released into the bloodstream and travel to the lung where they cause severe lung inflammation and hypoxemia [13-15]. Secretory phospholipase A2 is thought to convert bone marrow phospholipids to free fatty acids, which initiate an inflammatory response and lung injury similar to that triggered by intravenous administration of oleic acid in mouse models of the acute respiratory distress syndrome [16]. A more recent review based on the triolein-induced model of fat embolism has postulated that there is a vicious cycle involving oleic acid derived from embolized triglyceride, and angiotensin II (both of which are pulmonary toxicants) [17]. The lipid increases angiotensin generation and angiotensin increases triglyceride lipolysis [17].

Oil-Red-O staining of lipid accumulations within alveolar macrophages is diagnostic of the fat emboli syndrome and the lipid accumulations can be identified in more than 16% of cases of acute chest syndrome. One retrospective/prospective review of 21 autopsy cases that included Oil-Red-O and elastic staining of lung tissue from patients with SCD between 1990 and 2004 showed higher-than-expected percentages of acute and chronic sickle cell-related lung injury such as fat embolism which was 33.3% [18].

Standard preventive and therapeutic options for ACS are mostly conservative: cautious hydration, oxygen therapy, pain control, antibiotics, simple and exchange transfusion, and incentive spirometry [15]. Hydroxyurea is the first clinically acceptable drug shown to prevent painful crises and reduce the incidence of acute chest syndrome in adults with sickle cell anemia but does not have a role in the treatment of crises or chest syndrome in progress [19]. 

In our study, we consider the relationship between the pathophysiology of PFE and ACS. In a rat model of PFE, the injection of neutral lipid triolein as a surrogate for bone marrow fat led to deleterious changes in pulmonary vasculature [20-21]. Lung injury from PFE leads to regional hypoxemia which leads to increased deoxygenation of hemoglobin S followed by hemoglobin polymerization and vaso-occlusion which in turn promote bone marrow infarction and pulmonary vaso-occlusion perpetuating the vicious cycle of ACS in SCD. Furthermore, chronic alveolar hypoxemia stimulates the renin-angiotensin-aldosterone system (RAS) and this system may be intricately involved in the changes in pulmonary vascular structure and the stress response to the acute lung injury [22-25]. Some of these changes were mitigated by the administration of the ACEI, captopril, and ARB, losartan [26-27]. The damage caused by vaso-occlusive crises and ACS in SCD in some manner originate from free radical oxidant stress and activation of vascular oxidases [28-30]. The damage from PFE also occurs from the direct toxic effect of oleic acid formed by the action of different pulmonary lipases to fat in the pulmonary vasculature [20]. The microvascular changes in PFE can also be described in SCD pain crises or ACS. The relief of these changes by ACEI and ARB as demonstrated by the above-mentioned animal studies suggest a role for ACEI and ARB in the prevention of ACS [24]. Later studies have added the direct renin inhibitor, aliskiren, to the list of RAS modifiers for this protective effect [21]. Based upon the above pathophysiology, we hypothesized that the time to readmission after one hospitalization for ACS will be reduced in patients taking ACEI/ARB compared to patients not on these medications. The results of this study are significant because they would provide insight into a potential treatment or prevention for recurrent ACS.

This article was previously presented as a meeting abstract at the May 2018 CHEST International Meeting.

## Materials and methods

Study population

This study was designed as a retrospective cohort study. Inclusion criteria were adults (age 18 to 100 years) with sickle cell anemia (HbSS), hemoglobin SC (HbSC) disease, sickle cell thalassemia (HbSβThal), and ACS over 16 years (January 1, 2000, to March 31, 2016). Exclusion criteria were children (<18 years old), elderly adults (>100 years old), and patients with sickle cell trait. The case population consisted of patients with HbSS, HbSC, or HbSβThal who also reported taking ACEI or ARB. The control population was patients with HbSS, HbSC, or HbSβThal who did not report taking ACEI or ARB.

Data collection

Data for this study were collected from the Health Facts database, a large database that contains de-identified information from the electronic medical records of United States hospitals in which Cerner Corporation© has a data use agreement. Cerner © has established HIPAA-compliant operating policies to establish de-identification. Relevant diagnostic information was obtained through specific search-box queries. We determined the proportion of patients in the case and control populations who developed initial or subsequent ACS. Specifically, we searched within the database for patients’ reported presentation of ACS in the 16 years outlined above. To determine whether patients took ACEI/ARB, all types and dosages of ACEI or ARB were plugged into the Health Facts search. We assumed that if a subject had an ACEI or an ARB on their medication list, they were compliant with the medication. To further explore the impact of certain patient characteristics, such as age, gender, and smoking history on the relationship between ACEI/ARB use and ACS, these traits were also included in our search through the Health Facts database. Other characteristics such as body mass index (BMI) and hydroxyurea did not yield enough results from the database to contribute significant power to the study, so these results are withheld.

Statistical analysis

Kaplan-Meier estimates explored a time-to-event model of ACS readmission for all patients in the case and control groups. Length of stay (LOS) and overall survival (OS) were also calculated. Multivariable analysis using Cox proportional hazards regression was used to determine the effect of ACEI/ARB therapy on the risk for ACS, adjusting for the effects of age, gender, and smoking history. The primary outcome measure of this study was readmission rates for ACS after one hospitalization in patients who reported taking ACEI/ARB compared to patients who did not take ACEI/ARB, given as an odds ratio (OR). The dose and type of ACEI/ARB, as well as length of treatment with ACEI/ARB, was not factored into the analysis. The secondary outcome measure of this study was the effect of three covariates (age, gender, smoking) on readmission rates for patients who did take ACEI/ARB, given as an adjusted hazards ratio. Results were reported around a 95% confidence interval.

## Results

Our study yielded 6972 patients in total. Of which, 667 patients (9.6%) reported taking ACEI/ARB. The average age was 38 years, range 18 to 90 years. Most patients were female (n = 4366/6969, 63%, with three missing values). Only 16% reported smoking (n = 1132). Interestingly, of the patients who reported their race, almost all were African American (n = 6185/6838, 90%, with 134 missing values). The patients who reported ACEI/ARB use tended to be older (54 +/- 17 years) but only 26% reported also smoking (n = 174) (Table 1).

**Figure 1:**
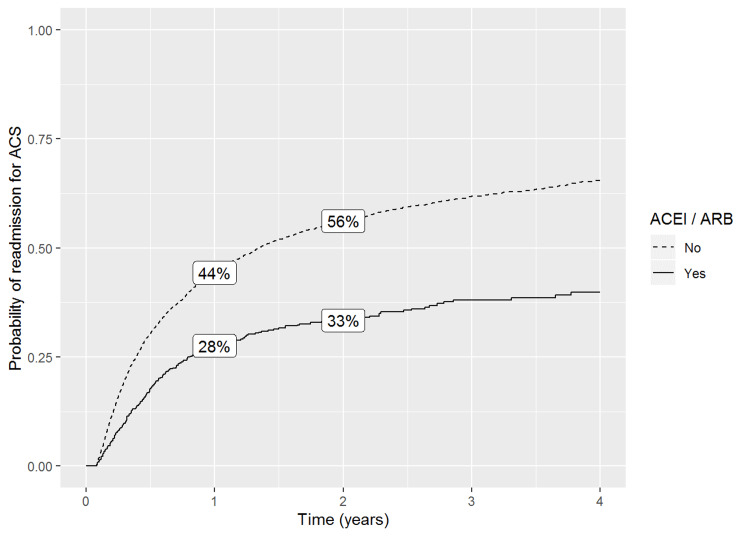
Probability of readmisson for acute chest syndrome (ACS) ACS: Acute chest syndrome, ACEI: Angiotensin-converting enzyme inhibitors, ARB: Angiotensin receptor blockers

Our primary outcome measure resulted in readmission rates being higher for patients not taking ACEI/ARB than those who did: OR 0.44 (95% CI, 0.43 to 0.46) versus OR 0.28 (95% CI, 0.24 to 0.31) at one year (Figure 1). A similar pattern was seen when comparing readmission rates for these groups at two years: OR 0.56 (95% CI, 0.55 to 0.58) versus OR 0.33 (95% CI, 0.29 to 0.37). Interestingly, patients who reported ACEI/ARB use had a longer length of stay (7 +/-9) than patients who did not (4 +/- 11; n = 6302 with three missing values). Analyzation of our secondary outcome measure via the covariate analysis revealed that age had the strongest effect on readmission rates for patients taking ACEI/ARB with an adjusted HR of 0.78 (95% CI, 0.68 to 0.91). Gender had the highest rate of readmission of all three covariates, with a higher rate of readmission for males (adjusted HR 1.32, 95% CI 1.23, 1.42).

## Discussion

Despite an increased understanding of the pathophysiology of SCD, the treatment options for disease-related complications, including ACS, remain limited. Hydroxyurea was approved by FDA in 1998. It is the first pharmacological agent to improve outcomes in patients with sickle cell anemia, such as its effect in decreasing the incidence of ACS. Hydroxyurea mainly exerts its effect by increasing fetal hemoglobin. Hydroxyurea is oxidized by heme groups to produce nitric oxide (NO) to form NO metabolites and also has shown to reduce expression of vascular adhesion molecule-1 (VCAM-1) which may contribute to its efficacy even before its effect on fetal hemoglobin [31-35]. However, it does not have a role in the treatment of ongoing vaso-occlusive crises or acute chest syndrome [19].

More recently, the P-selectin inhibitor crizanlizumab was associated with a significant lowering of the frequency of SCD-related pain crises compared to placebo but did not show a significant difference for ACS [36]. Oral administration of pharmaceutical-grade L-glutamine in children and adults with SCD reduced the number of pain crises and ACS compared to placebo with or without hydroxyurea [37]. L-glutamine exerts its effect primarily by raising the nicotinamide adenine dinucleotide + hydrogen (NAD+/NADH) reduction-oxidation ratio within sickle cells, creating an environment of less oxidative stress [38].

Studies have shown PFE from bone marrow fat causes a severe and distinct form of ACS in SCD [39]. In animal models, drugs interfering with the RAS namely, ACEI captopril, ARB losartan, and the direct renin inhibitor aliskiren have shown to have a protective effect on the pulmonary vasculature in PFE [26]. In both studies, the interference of RAS reduced the inflammatory, vasoconstrictor and profibrotic effects at 48 hours in the lungs for fat embolism (FE) syndrome besides the vascular lumen remaining patent, and the reduction in size and number of fat droplets [21]. The ARB losartan was also shown to block the late phase of fat embolism beyond six weeks indicating involvement of RAS in late as well as early stages of histopathological changes following fat embolism [40]. The pulmonary pathology in this animal model was found to be parallel to that found in a fatal case of fat embolism syndrome in a patient who developed respiratory failure after caesarean delivery [41-42]. These results suggested that the use of drugs that act on RAS might be an effective and targeted therapy for ACS [43].

Our study is a retrospective cohort study. We show that the readmission rate for ACS after one hospitalization is lower for patients who reported taking ACEI/ARB compared to those who did not. This effect is consistent with the animal model findings that these agents which interfere with RAS, mitigate the detrimental changes to pulmonary vasculature of PFE. Henceforth, by a similar mechanism, they could mitigate detrimental changes in pulmonary vasculature in ACS in SCD. Interestingly, age had the most impact on lowering readmission rates for patients with ACS. Older patients had lower readmission rates than younger patients. This could be due to the heterogeneity in phenotype seen in SCD. Perhaps patients who are older tend to have less severe disease and thus have fewer hospitalizations than younger patients with a more aggressive disease. Hypertension is a disorder common to middle-aged and older patients. It can be presumed that these patients are more likely to take ACEI/ARB (our study found that the average age for ACEI/ARB users was 54 years), and thus benefit from the therapeutic effects of these medications more than younger patients who tend not to be on them.

The study is limited by its retrospective nature in demonstrating a correlation between ACEI/ARB use and a decrease in readmission rates. Additionally, there is an assumption of patient compliance with ACEI/ARB. We were unable to identify the reason patients were taking ACEI/ARB, how long they were taking the medications, and determine compliance with the medications. This study does suggest a correlation between decreased readmission rates for ACS and the use of ACEI/ARB that may serve to guide the management of patients with SCD. Further study goals would involve prospective clinical trials comparing ACS readmission in patients who receive ACEI/ARB/renin inhibitor therapy versus placebo after an initial event to confirm this preventative effect. The possible future therapeutic use of these drugs after symptoms of ACS have developed needs to be investigated.

## Conclusions

Patients with SCD who reported taking ACEI or ARB had lower readmission rates for ACS; age was the strongest covariate. Our results may have a significant impact on the prevention of ACS. Prospective studies comparing ACEI or ARB therapy versus placebo are needed to confirm this preventative effect. The significance of our project will be to provide a potential treatment or a preventative strategy for ACS in patients with SCD through the administration of drugs that interfere with the RAS system such as ACEI or ARB or renin inhibitor. These drugs have a low toxicity profile and are already widely used to treat other cardiovascular disorders, such as hypertension. Both ACEI and ARB, and renin inhibitor could represent another group of medications that could potentially be disease-modifying for SCD and ACS where currently there are limited options. Hydroxyurea and long term transfusion are the only two widely used therapeutic options. The study is also important because ACS remains a major SCD-related complication that directly impacts mortality.
